# Molecular profiling and combinatorial activity of CCT068127: a potent CDK2 and CDK9 inhibitor

**DOI:** 10.1002/1878-0261.12148

**Published:** 2018-01-28

**Authors:** Steven R. Whittaker, Clare Barlow, Mathew P. Martin, Caterina Mancusi, Steve Wagner, Annette Self, Elaine Barrie, Robert Te Poele, Swee Sharp, Nathan Brown, Stuart Wilson, Wayne Jackson, Peter M. Fischer, Paul A. Clarke, Michael I. Walton, Edward McDonald, Julian Blagg, Martin Noble, Michelle D. Garrett, Paul Workman

**Affiliations:** ^1^ Cancer Research UK Cancer Therapeutics Unit Division of Cancer Therapeutics The Institute of Cancer Research London UK; ^2^ Northern Institute for Cancer Research University of Newcastle upon Tyne Medical School Newcastle upon Tyne UK; ^3^ Cyclacel Ltd. Dundee UK; ^4^ Present address: Samuel Lister Academy Bingley West Yorkshire BD16 1TZ UK; ^5^ Present address: School of Pharmacy and Centre for Biomolecular Sciences University of Nottingham, University Park Nottingham NG7 2RD UK; ^6^ Present address: School of Biosciences University of Kent Canterbury Kent CT2 7NJ UK

**Keywords:** ABT263, CCT068127, CDK, MCL1, seliciclib

## Abstract

Deregulation of the cyclin‐dependent kinases (CDKs) has been implicated in the pathogenesis of multiple cancer types. Consequently, CDKs have garnered intense interest as therapeutic targets for the treatment of cancer. We describe herein the molecular and cellular effects of CCT068127, a novel inhibitor of CDK2 and CDK9. Optimized from the purine template of seliciclib, CCT068127 exhibits greater potency and selectivity against purified CDK2 and CDK9 and superior antiproliferative activity against human colon cancer and melanoma cell lines. X‐ray crystallography studies reveal that hydrogen bonding with the DFG motif of CDK2 is the likely mechanism of greater enzymatic potency. Commensurate with inhibition of CDK activity, CCT068127 treatment results in decreased retinoblastoma protein (RB) phosphorylation, reduced phosphorylation of RNA polymerase II, and induction of cell cycle arrest and apoptosis. The transcriptional signature of CCT068127 shows greatest similarity to other small‐molecule CDK and also HDAC inhibitors. CCT068127 caused a dramatic loss in expression of DUSP6 phosphatase, alongside elevated ERK phosphorylation and activation of MAPK pathway target genes. MCL1 protein levels are rapidly decreased by CCT068127 treatment and this associates with synergistic antiproliferative activity after combined treatment with CCT068127 and ABT263, a BCL2 family inhibitor. These findings support the rational combination of this series of CDK2/9 inhibitors and BCL2 family inhibitors for the treatment of human cancer.

**Abbreviations**
BrdU: bromodeoxyuridine
CDK: cyclin‐dependent kinase inhibitor
CDKI: cyclin‐dependent kinase inhibitor
CI: combination index
GI_50_: 50% growth inhibition
HDAC: histone deacetylase
IAP: inhibitor of apoptosis
MAPK: mitogen‐activated protein kinase
RT‐qPCR: real‐time quantitative polymerase chain reaction
SDS/PAGE: sodium dodecyl sulfate/polyacrylamide gel electrophoresis
siRNA: short interfering ribonucleic acid

## 1 Introduction

Loss of cell cycle control is a hallmark of human cancer, and this can be achieved by overexpression of the CDK partner cyclins or by decreased expression or mutation of CDK inhibitors (CDKIs) from the INK4 and CIP/KIP families (Malumbres and Barbacid, 2009). For example, amplification of the 19q12 locus has been reported in up to 15% of estrogen receptor (ER)‐negative breast cancers, typically resulting in amplification of *CCNE1* (cyclin E1) (Adélaïde *et al*., 2007; Natrajan *et al*., 2012). The G_1_‐to‐S‐phase transition is regulated by CDK4/cyclin D and CDK6/cyclin D. CDK2/cyclin A, CDK2/cyclin E, and CDK1/cyclin A mediate progression through S phase to G_2_ and CDK1/cyclin B activity is involved in the initiation of mitosis (Malumbres and Barbacid, 2009). Importantly, CDK activities converge to phosphorylate the RB protein that functions to suppress the activity of E2F‐1, which in turn is required to stimulate the transcription of genes required for DNA synthesis. Current evidence supports a model whereby cumulative phosphorylation of RB displaces bound histone deacetylase (HDAC) and E2F‐1, relieving transcriptional repression and permitting gene transcription from E2F‐1‐dependent promoters (Zhang *et al*., 2000).

Targeting of CDK2/cyclin E activity by RNA interference or by small molecules is effective against human breast cancer cell lines exhibiting *CCNE1* amplification (Natrajan *et al*., 2012). CDKs are not only involved in orchestrating the correct passage through the cell cycle, but also key mediators of transcriptional control, in part by the regulation of RNA polymerase II (Bregman *et al*., 2000). CDK7/cyclin H and CDK9/cyclin T phosphorylate RNA polymerase II on its C‐terminal, which is permissive for initiation and elongation of nascent mRNA transcripts. Interestingly, a number of transcripts involved in cell cycle control and apoptosis are highly responsive to alterations in transcriptional regulation, the mRNAs having short half‐lives, and are thus rapidly lost following inhibition of transcription (Lam *et al*., 2001). Hence, some CDK inhibitors are capable of suppressing the transcription of key genes such as *MCL1*,* MYC*,* CCNB2* (cyclin B2), and several IAP family members (*BIRC1*,* BIRC2,* and *BIRC3*). Therefore, CDK inhibitors have the potential to perturb cell cycle progression through direct catalytic inhibition of CDK/cyclin complexes, loss of expression of the partner cyclins, and also sensitize cells to proapoptotic stimuli.

While it has been reported that CDKs can be dispensable for survival or cell cycle progression, it appears that this is due to a high degree of redundancy among the CDKs and their partner cyclins. For example, CDK2 knockout in mice proved to be nonlethal and RNAi‐mediated suppression of CDK2 did not affect cell proliferation (Ortega *et al*., 2003; Tetsu and McCormick, 2003). However, CDK1 is essential for survival in mice (Diril *et al*., 2012) and dual suppression of CDK1 and CDK2 presumably overcomes any redundancy in CDK2 function, resulting in cell cycle arrest and/or apoptosis (L'Italien *et al*., 2006). Combinatorial targeting of multiple CDKs therefore remains an attractive approach for cancer therapy (Whittaker *et al*., 2017).

We embarked upon a drug discovery program to develop CDK2 and 9 inhibitors that exhibited significantly improved pharmacological properties compared with the parental clinical drug seliciclib (CYC202, R‐roscovitine) (McClue *et al*., 2002; Raynaud *et al*., 2005; Whittaker *et al*., 2004). To this end, we identified CCT068127, a novel trisubstituted purine with improved potency, selectivity, metabolic stability, and antitumor activity compared with seliciclib (Wilson *et al*., 2011). Here, we describe the biochemical and cellular characterization of CCT068127 and identify synergistic drug combinations to further improve the efficacy of such CDK2/9 inhibitors for the treatment of colorectal cancer.

## 2 Materials and methods

### 2.1 Cell culture and reagents

All human cancer cell lines (HT29, colorectal adenocarcinoma; HCT116, colon carcinoma; COLO205, colon adenocarcinoma; RKO, colon carcinoma; SKMEL28, malignant melanoma; WM266.4, malignant melanoma) were obtained from the American Type Culture Collection and grown in the recommended culture medium, supplemented with 10% FBS, at 37 °C and an atmosphere of 5% CO_2_. Cell lines were passaged for less than 6 months upon receipt. CCT068127 was synthesized as described (Wilson *et al*., 2011) and solubilized in dimethyl sulfoxide to a stock concentration of 10 mmol·L^−1^. The BCL2 family inhibitor ABT263 was purchased from Selleck Chemicals (Tse *et al*., 2008). The ERK inhibitor VTX‐11e was purchased from Tocris (Aronov *et al*., 2009).

### 2.2 *In vitro* biochemical kinase assays

Profiling of CCT068127 against a panel of ~ 30 recombinant human kinases was performed as previously described (McIntyre *et al*., 2010; Wang *et al*., 2004; Wilson *et al*., 2011).

### 2.3 Enzyme expression and purification

The gene encoding full‐length human CDK2 was cloned into the pGEX‐6P‐1 and transformed into BL21(DE3)pLysS cells (Promega, Southampton, UK). Cultures were grown for 2–3 h at 37 °C, and then, the temperature was decreased to 18 °C prior to induction with 0.25 mmol·L^−1^ IPTG at OD_600 _= 0.6. The cultures were allowed to grow for an additional 20–24 h at 18 °C and were harvested by centrifugation. All purification steps were performed by FPLC at 4 °C. Harvested cells were resuspended in 50 mmol·L^−1^ HEPES buffer (pH 7.5) containing 150 mmol·L^−1^ NaCl, 2 mmol·L^−1^ DTT, and 0.5 mg·mL^−1^ lysozyme at 4 °C for 1 h. After sonication and centrifugation (1 h at 29 000 ***g***), the supernatant was purified by immobilized glutathione Sepharose chromatography (GE LifeSciences, Little Chalfont, UK). Following incubation of peak fractions with 3C protease (20 : 1) at 4 °C, the cleaved GST‐tag was separated by size exclusion chromatography using a Superdex 75 (26/60) column and eluted with 50 mmol·L^−1^ HEPES buffer (pH 7.4) containing 150 mmol·L^−1^ NaCl and 1 mmol·L^−1^ DTT. Purified CDK2 was buffer‐exchanged into 100 mmol·L^−1^ Na/K phosphate buffer (pH 7.4) containing 1 mmol·L^−1^ DTT and concentrated to 10 mg·mL^−1^ for crystallization.

### 2.4 X‐ray crystallography

Crystallization was performed at 20 °C using the sitting drop vapor diffusion method. Crystals of human CDK2 were grown from 0.1 m HEPES pH 7.5 and 10% PEG3350 over a reservoir of 50 mmol·L^−1^ HEPES pH7.5 and 50 mmol·L^−1^ Na/K phosphate buffer (pH 7.4). Crystals were harvested in cryoprotectant containing 1 mmol·L^−1^ CCT068127 (50 mmol·L^−1^ HEPES pH 7.5, 50 mmol·L^−1^ Na/K phosphate buffer (pH 7.4), 15% PEG3350, 25% (v/v) ethylene glycol, 1% DMSO, and 1 mmol·L^−1^ CCT068127) 24 h prior to data collection. X‐ray diffraction data were recorded at Diamond Light Source (Harwell, Oxfordshire, UK). Data processing was carried out using XDS, POINTLESS/AIMLESS (Evans, 2011), and other programs of the CCP4i suite (Collaborative Computational Project, 1994) run through the CCP4i2 GUI. The structure was solved by molecular replacement using PHASER (McCoy *et al*., 2007) and pdb 3QXP as a starting model. REFMAC (Murshudov *et al*., 1997) was employed for refinement, and model building was performed using COOT (Emsley *et al*., 2010). Figures were prepared using PYMOL (Schrödinger, LLC, Cambridge, UK). The coordinates of the CDK2‐CCT068127 crystal structure have been deposited in the Protein Data Bank with accession code 5MHQ.

### 2.5 Cell proliferation

The effect of CCT068127 and seliciclib on cancer cell proliferation was measured using the sulforhodamine B (SRB) assay as described previously (Whittaker *et al*., 2004). For long‐term colony formation assays, HT29 colon cancer cells were seeded into 12‐well plates and then treated with the inhibitors for 5 days. Compounds were then washed away and the cells were allowed to proliferate for a further 7 days. After this, cells were fixed with 4% formaldehyde for 30 min, and cellular protein and DNA were stained with 0.5% crystal violet solution for 30 min and washed in water to remove excess dye. The colony plates were imaged on a FluorChem E System (ProteinSimple, Oxford, UK). Bound dye was subsequently resolubilized in 10% acetic acid and absorbance measured at 595 nm to quantify cell number.

### 2.6 Western blotting

Cells were seeded at a density of approximately 4 × 10^5^ cells/well in 6‐well plates and incubated overnight prior to drug treatment. They were then harvested in NP‐40 buffer as previously described (Whittaker *et al*., 2004) and lysates were normalized using the Bradford assay (Bio‐Rad, Watford, UK). Equal amounts of protein were resolved by SDS/PAGE using 4–12% bis‐tris gels (or 6% tris‐glycine gels to demonstrate a mobility shift in RB in Fig. 3D) and transferred to nitrocellulose membranes (Life Technologies, Paisley, UK). The antibodies used in this study are described in Table S1. Primary antibodies were labeled with fluorescently labeled IRDye secondary antibodies (Li‐Cor, Cambridge, UK), and proteins were visualized using an Odyssey scanner (Li‐Cor).

### 2.7 Cell cycle effects

Cell cycle analysis was performed as previously described (Whittaker *et al*., 2004). Briefly, for cell cycle distribution profiles, cells were exposed to inhibitors for the indicated times, and then attached cells were harvested by trypsinization. Cells were washed in PBS and fixed in ice‐cold 70% ethanol. Cellular DNA was stained using propidium iodide and analyzed by flow cytometry. To determine the number of cells undergoing DNA synthesis, cells were pulsed with 10 μmol·L^−1^ bromodeoxyuridine (BrdU, Sigma, Gillingham, UK) for 30 minutes prior to harvesting. Cells were fixed in 70% ethanol and nuclei were isolated using 2 mol·L^−1^ HCl and 0.2 mg·mL^−1^ pepsin. Incorporated BrdU was detected using an anti‐BrdU antibody coupled to FITC (Molecular Probes, Paisley, UK), and cells were quantified by flow cytometry.

### 2.8 Gene expression analysis

Cells were exposed to the inhibitors for 24 h prior to RNA extraction with Trizol (Invitrogen, Paisley, UK) and subsequent mRNA purification by Oligotex (Qiagen, Manchester, UK). Eight microgram of mRNA was labeled with either Cy3 or Cy5 dCTP via RT‐PCR and the labeled probe was then purified and 2 μg of each sample was hybridized to a custom cDNA array of 5808 genes spotted onto poly‐lysine‐coated glass slides at 65 °C overnight. Slides were scanned using a GenePix 4000B (Axon Labs, Union City, CA, USA) and spots verified using genepix software. Data analysis was performed in GeneSpring (Silicon Genetics, Redwood City, CA, USA). Gene expression data were analyzed relative to the DMSO controls by comparative marker selection using a *t*‐test to rank expression changes in Gene‐E (http://www.broadinstitute.org/cancer/software/GENE-E/). *A* ≥ twofold change in expression was used to refine the selection criteria. Gene expression profiles were analyzed using Connectivity Map (Lamb *et al*., 2006) and STRING databases (Franceschini *et al*., 2013).

For RT‐qPCR assays, mRNA was isolated from cancer cells using RNeasy Mini kit (Qiagen) according to the manufacturer's recommendations. Extracted RNA was used to generate cDNA with the High Capacity cDNA Reverse Transcription kit (Applied Biosystems, Paisley, UK). Primer combinations for the respective genes were designed according to the Harvard Primer Bank (http://pga.mgh.harvard.edu/primerbank) and are listed in Table S2. Gene expression was analyzed using Power SYBR Green gene expression assays (Life Technologies). Quantitative PCR was carried out in triplicate on the ViiA 7 Real‐Time PCR System (Applied Biosystems).

### 2.9 siRNA transfection

HT29 cells were reverse‐transfected in 6‐well or 96‐well plates using 0.4% RNAiMAX (Life Technologies) transfection reagent and 25 nm of ON‐TARGETplus siRNA (Dharmacon, Cambridge, UK) in OptiMem. Cells were then cultured for 4 days and analyzed as required. A nontargeting siRNA (siCTRL) and four different siRNAs targeting CDK5 were used (siCTRL: D‐001810‐01‐05; siCDK5: J‐003239‐9, J‐003239‐10, J‐003239‐11, J‐003239‐12).

### 2.10 Statistical analysis

For statistical analysis of protein expression data, the mean of three independent repeats per condition was used for two‐tailed *t*‐tests to determine significance of change, relative to the appropriate control. This was deemed the most appropriate test as nonparametric tests such as the Mann–Whitney *U*‐test require greater sample numbers to enable significance testing, as discussed by Krzywinski and Altman (2014).

## 3 Results

### 3.1 CCT068127 is a potent and stable inhibitor of CDK2 and 9

We previously reported a medicinal chemistry campaign to generate more potent, selective, and metabolically stable analogs of the clinical cyclin‐dependent kinase inhibitor seliciclib (CYC202, R‐roscovitine) (Wilson *et al*., 2011). CCT068127 was identified as having significantly increased potency toward human CDK1/cyclin B (15‐fold), CDK2/cyclin E (22‐fold), CDK5 (15‐fold), and CDK9/cyclin T (11‐fold) kinase activities, when compared to the parental seliciclib (Table**** 1). Importantly, selectivity toward CDK2 and 9 versus CDK4 and CDK7 is enhanced compared to seliciclib, as the concentration required to inhibit kinase activity by 50% (IC_50_) remained relatively unchanged for CDK4/cyclin D and CDK7/cyclin H. Although the potency of CCT068127 for CDK1 is increased over seliciclib, the IC_50_ is still relatively high at 1.12 μm. Overall, CCT068127 showed greatest *in vitro* potency against CDK2, CDK5, and CDK9.

**Table 1:** *In vitro* kinase inhibition by CCT068127 and seliciclib. Compounds were tested against human recombinant enzymes *in vitro* as previously described (McIntyre *et al*., 2010; Wang *et al*., 2004; Wilson *et al*., 2011) except for CDK5/p35, which was carried out using the Invitrogen Select Screen service. Results are the mean of three independent repeats ± SE except for CDK5/p35, which is the mean of two determinations. The following kinases had IC_50_ values greater than 10 μmol·L^−1^ for both compounds: ABL, AKT, CAMKII, CK2, ERK2, GSK3, PKA, PKC, PLK1, S6, SAP2KA, AURKA, FLT3, SRC, LCK, PDGFB, AURKB, VEGFR1, VEGFR2, and IKKA. Data for seliciclib were reported previously (Mariaule and Belmont, 2014; Wilson *et al*., 2011)

Kinase assay	CCT068127 Mean IC_50_ (μmol·L^−1^) ± SE	Seliciclib IC_50_ (μmol·L^−1^)
CDK1/B	1.1 ± 0.29	17
CDK2/A	0.11 ± 0.02	ND
CDK2/E	0.010 ± 0.001	0.22
CDK4/D1	4.8 ± 0.6	27
CDK5/p35	0.07	1.0
CDK6/D3	6.2 ± 0.6	ND
CDK7/H	0.52 ± 0.09	0.52
CDK9/T	0.09 ± 0.03	0.80

CCT068127 displays notably improved cellular activity compared with seliciclib, with the average concentration to reduce cell proliferation by 50% (GI_50_) in a panel of human colon cancer and melanoma cell lines being 0.5 μmol·L^−1^ versus 12 μmol·L^−1^, respectively. This 20‐fold increase in cellular potency was consistent with the enhanced activity against CDK2, CDK5, and CDK9 (Table**** 2). In addition, we have previously reported that CCT068127 displays greater metabolic stability than seliciclib (Wilson *et al*., 2011).

**Table 2:** Antiproliferative activity of CCT068127 and seliciclib in human colon cancer and melanoma cell lines. GI_50_ values for cell proliferation were determined in human colon cancer and melanoma cell lines following exposure for four population doublings using SRB analysis as the endpoint (Whittaker *et al*., 2004). Values reported are the mean of three independent repeats ± SE. ND, not determined

Cell line	Tissue	CCT068127 GI_50_ (μmol·L^−1^) ± SE	Seliciclib GI_50_ (μmol·L^−1^) ± SE
HT29	Colorectal adenocarcinoma	0.85 ± 0.03	15 ± 3.4
HCT116	Colon carcinoma	0.25 ± 0.03	6.4 ± 1.8
COLO205	Colon adenocarcinoma	0.50 ± 0.09	11 ± 1.2
RKO	Colon carcinoma	0.66 ± 0.03	10 ± 0.26
SKMEL28	Malignant melanoma	0.31 ± 0.07	8.5 ± 2.1
WM266.4	Malignant melanoma	0.40 ± 0.06	18 ± 2.4
Average	–	0.5	12

### 3.2 Crystal structure of CCT068127 bound to CDK2

**Figure 1:**
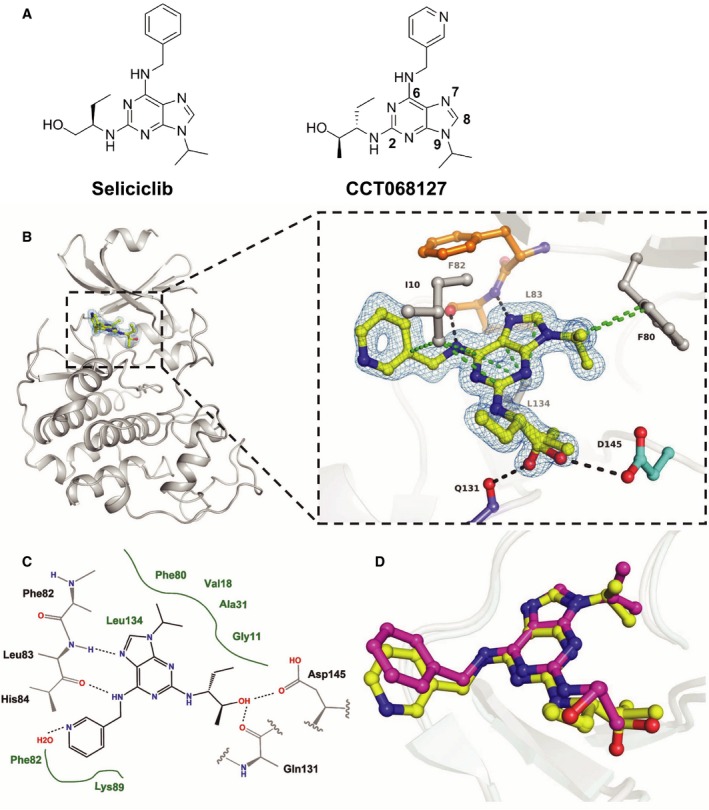
Enhanced potency of CCT068127 over seliciclib is achieved by additional ligand interactions with CDK2 and CDK9. (A) Chemical structures of seliciclib and CCT068127; numbering of the purine scaffold is indicated for CCT068127. (B) Secondary structure representation of human CDK2 in complex with CCT068127 determined by X‐ray crystallography at 1.3 Å resolution. The inset shows the binding interactions of CCT068127 within the ATP binding pocket. The hinge region is indicated in orange, Asp145 of the DFG motif in cyan, and CCT068127 in yellow. Displayed in blue is the 2Fo‐Fc electron density, contoured at 1σ around CCT068127. The data and refinement statistics are shown in the Supporting information (Table S3). The hydrogen bonding and van der Waals (hydrophobic) interactions are shown as black and green dotted lines, respectively. PDB: 5MHQ. (C) Schematic presentation of the binding interactions between CCT068127 and CDK2. (D) Alignment of CCT068127 (yellow) and seliciclib (magenta) (PDB: 3DDQ) bound to CDK2.

Based on structure–activity relationships for synthesized analogs (Wilson *et al*., 2011), we hypothesized that the more potent inhibition of CDK2 and CDK9 for CCT068127 in comparison with seliciclib (Table**** 1) is due to the presence of the pyridyl nitrogen and the methyl group of defined stereochemistry in the hydroxyalkyl side chain of CCT068127 (Fig. 1A). Our initial *in silico* docking suggested that, compared to seliciclib, the hydroxyl group of CCT068127 could form an additional hydrogen bond with Asp145 of the DFG motif in human CDK2 (data not shown).

To determine the precise binding mode, we determined the X‐ray crystal structure of CCT068127 with CDK2 (Fig. 1B). The CCT068127–CDK2 complex formed crystals with space group P2_1_2_1_2_1_ that diffracted to 1.3Å resolution. The structure was refined to R_factor_ and R_free_ values of 19.6% and 23.4%, respectively (Table S3). This shows that CCT068127 acts as a type I kinase inhibitor of CDK2 and binds to the hinge region of the ATP binding pocket through two hydrogen bonds, formed by the purine N7 and the exocyclic nitrogen at C6 of the purine scaffold that interact with the main chain amine and carbonyl of Leu83, respectively (Fig. 1B,C). In addition, our high‐resolution electron density map of the inhibitor–CDK2 complex has allowed unambiguous assignment of the conformation of the hydroxymethyl moiety. As predicted by our modeling, the side chain hydroxyl forms an additional hydrogen bond with the carboxylic acid side chain of Asp145, part of the DFG motif. Electron density indicates two alternate conformations for the hydroxyl group, with the minor pose forming an interaction with the main chain carbonyl of Gln131. Also CCT068127 forms multiple van der Waals (hydrophobic) interactions with surrounding residues (3.4Å < *d* < 4.0Å). The purine core is sandwiched between Ile10 and Leu134, with the N9‐isopropyl moiety stacking up against the gatekeeper residue Phe80.

Superimposition of CDK2 structures bound to CCT068127 and seliciclib illustrates a key rotation of the C2 substituent that orients the pendant hydroxyl moiety toward Asp145. The additional hydrogen bound formed in this altered pose provides a plausible explanation for the increased potency CCT068127 relative to seliciclib (Fig. 1D). The pyridine moiety of CCT068127 is slightly shifted relative to the phenyl group of seliciclib (Fig. 1D) possibly resulting from the engagement of the pyridine nitrogen in water‐mediated hydrogen bonds with CDK2.

### 3.3 Inhibition of CDK2 and CDK9 activity correlates with decreased substrate phosphorylation in cells

**Figure 2:**
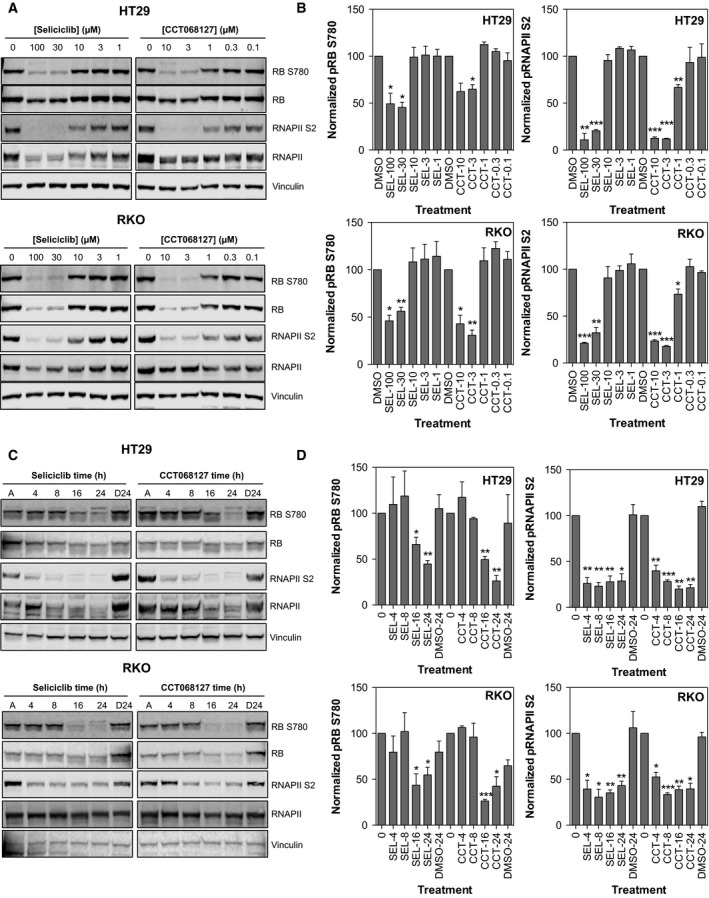
CCT068127 potently inhibits RB and RNA polymerase II phosphorylation in human cancer cells. (A) HT29 and RKO colon cancer cells were treated with increasing concentrations of CCT068127 or seliciclib for 24 h. Cell lysates were analyzed by western blotting for the phosphorylation of RB (a measure of CDK2 inhibition) and phosphorylation of RNA polymerase II (a measure of CDK9 inhibition). (B) Quantification of RB S780 and RNAPII S2 phosphorylation in (A), normalized to total RB or total RNAPII, respectively, and expressed as a percentage of the DMSO control (mean percentage of control values presented from three independent repeats ± SE, significant difference from control indicated by *P < 0.05, **P < 0.01, ***P < 0.001, two‐tailed *t*‐test). (C) HT29 and RKO cells were treated with equiactive (3xGI_50,_ SRB assay) concentrations of CCT068127 (2.55 μm for HT29 and 1.98 μm for RKO) or seliciclib (45 μm for HT29 and 30 μm for RKO) for the indicated times. A: asynchronously growing, untreated cells; D24: cells treated with DMSO for 24 h. Cell lysates were analyzed by western blotting for the indicated proteins. (D) Quantification of RB S780 and RNAPII S2 phosphorylation in (C), normalized to total RB or total RNAPII, respectively, and expressed as a percentage of the time 0 control (mean percentage of control values presented from three independent repeats ± SE, significant difference from control indicated by *P < 0.05, **P < 0.01, ***P < 0.001, two‐tailed *t*‐test).

To confirm that CCT068127 retains activity against CDKs in cells, the phosphorylation of selected CDK substrates was determined. We used a cell‐based assay for protein phosphatase 1 phosphorylation to measure CDK1 activity following compound treatment. PP1 phosphorylation at T320 shows inhibition with an IC_50_ of 6 μm for CCT068127 and 71 μm for seliciclib, 4–9 times greater than the respective GI_50_ values (Fig. S1) (Kwon *et al*., 1997). Of note, cyclin B1 expression was reduced only at higher concentrations, with an IC_50_ of 15 μm for CCT068127 and >100 μm for seliciclib, suggesting that loss of cyclin B1 contributes to, but is not primarily responsible for, the antiproliferative activity of these compounds. Phosphorylation of the RB protein by CDKs facilitates DNA synthesis and cell cycle progression through the displacement of HDACs and E2F family transcription factors from RB (Brehm *et al*., 1998). The phosphorylation status of RB was therefore assessed following a 24‐h exposure to CCT068127 or seliciclib in HT29, RKO, and COLO205 human colon cancer cells (Figs 2A and S2A). We found that a concentration of 3 μmol·L^−1^ CCT068127 or greater reduces phosphorylation of RB at S780, whereas 30 μmol·L^−1^ seliciclib is required to elicit the same effect, consistent with at least a 10‐fold greater potency of CCT068127 over seliciclib in the biochemical assays with recombinant proteins. Notably, the inhibition of RB phosphorylation was only partial (~50%) as determined by quantification of the blots and was less pronounced in the COLO205 cells, due to the loss of total protein (Figs 2B and S2B).

In addition to effects on the cell cycle mediated through inhibition of RB phosphorylation, CDKs are known to regulate transcription through modification of RNA polymerase II, which is required for the generation of some transcripts arising from pol II‐dependent promoters (Oelgeschlager, 2002). Phosphorylation of RNA polymerase II at S5 and S2 is catalyzed by CDK7 and CDK9, respectively, although other CDKs have also been shown to target these sites (Jeronimo *et al*., 2016). Inhibition of these CDKs by compounds such as flavopiridol and seliciclib has been shown to have dramatic effects on the transcriptional machinery leading to a profound inhibition of transcription (Lam *et al*., 2001; Whittaker *et al*., 2004, 2007). RNA polymerase II phosphorylation was assessed at S2 following treatment with CCT068127 or seliciclib (Figs 2A and S2A). We found that inhibition of RNA polymerase II phosphorylation occurs following treatment with 3 μmol·L^−1^ or greater CCT068127. Seliciclib is less potent, again requiring approximately 30 μmol·L^−1^ to reduce RNA polymerase II phosphorylation to equivalent levels. This is likely in part due to the loss of total protein as previously observed (Whittaker *et al*., 2004). In contrast to RB, RNA polymerase II S2 phosphorylation was more completely inhibited (up to 80%) by both compounds (Figs 2B and S2B). Interestingly, we found that phosphorylation of S5 of RNA polymerase II was less sensitive to treatment and reflected loss of the total protein more than specific loss of phosphorylation (data not shown).

Time‐course experiments using a 3xGI_50_ concentration of both compounds were also performed in HT29 and RKO human colon cancer cells. Equiactive concentrations of each compound were used to enable a comparison of the molecular alterations induced by each compound at concentrations that have similar effects on cell proliferation/survival. We found that both compounds inhibit RB phosphorylation with similar kinetics from 16 to 24 h (Figs 2C,D and S2C,D). RNA polymerase II phosphorylation is inhibited more rapidly, with both compounds reducing phosphorylation at S2 from 4 to 24 h (Figs 2C,D, and S2C,D). As expected, the inhibition of RNA polymerase II phosphorylation is associated with decreased expression of the protein at later time points. These data demonstrate that loss of RNA polymerase II phosphorylation precedes loss of RB phosphorylation. To evaluate whether the expression of CDKs was being modulated by seliciclib and CCT068127, the levels of CDK1, 2, 5, and 9 were assessed following a 24‐h exposure to these compounds (Fig. S3A). The expression of these proteins was largely unaltered, with the exception of CDK9, which was decreased at 30–100 μm seliciclib and 3–10 μm CCT068127 in HT29 cells and at 100 μm seliciclib and 10 μm CCT068127 in RKO cells. The expression of cyclins E1, T1, B1, and D1 was also assessed and cyclin D1 levels were found to be decreased by CCT068127 and seliciclib (Fig. S3A); more modest decreases in cyclin T1 were observed with CCT068127. Notably, cyclin E1 expression was increased by seliciclib (10–30 μm) and by CCT068127 (0.3–1 μm) (Fig. S3A). This may reflect inhibition of CDK2, which is known to phosphorylate cyclin E1 and target it for degradation (Clurman *et al*., 1996), and has been reported as a marker of CDK2 inhibition (Sakurikar *et al*., 2016). We could not detect expression of the p35 regulatory subunit of CDK5, which is reportedly expressed primarily in postmitotic neurons, although a role in cancer is emerging (Shupp *et al*., 2017). To specifically address the effect of reduced CDK5 activity on cell proliferation, we used four different siRNAs to deplete HT29 cells of CDK5 by 80–90% (Fig. S4A). Under these conditions, we observed minor reductions of up to 10–20% in proliferation with two of the four siRNAs tested, with the remaining two siRNAs having no effect despite good knockdown of CDK5 expression (Fig. S4B). A time course of seliciclib and CCT068127 showed that the decrease in CDK9, cyclin T1, and cyclin D1 expression occurred at 16–24 h (Fig. S3B), whereas inhibition of RNA polymerase II phosphorylation was observed as early as 4 h (Fig. 2C,D). This is consistent with direct inhibition of CDK9 mediating the early effects of these compounds, although at later time points effects on transcription may also contribute to the loss of CDK activity, for example, by cyclin depletion.

### 3.4 Inhibition of cell cycle progression and DNA synthesis by CCT068127

**Figure 3:**
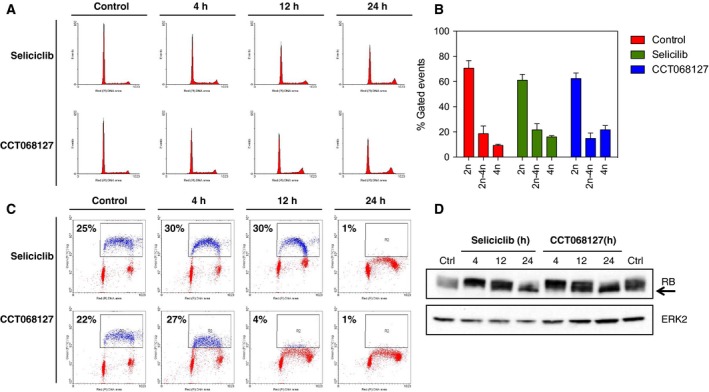
Induction of cell cycle arrest by CCT068127 and seliciclib. (A) HT29 human colon cancer cells were incubated with equiactive (3xGI_50_, SRB assay) concentrations of CCT068127 or seliciclib for 4‐24 h. Cell cycle distribution was assessed by propidium iodide staining and analysis by flow cytometry. (B) Quantification of cell cycle distribution profiles from (A). Data presented are the mean of three independent experiments ± SE. (C) HT29 cells were treated as in (A) and were pulse‐labeled with BrdU, then fixed, and stained with propidium iodide. BrdU incorporation was determined using a FITC‐conjugated antibody to BrdU and detected by flow cytometry. The percentage of BrdU positive cells is indicated. (D) HT29 cells were treated as in (A) and cell lysates were analyzed by western blotting for the indicated proteins. The arrow indicates hypophosphorylated RB.

To investigate the cell cycle effects of CCT068127, HT29 colon cancer cells were exposed to the respective 3xGI_50_ concentrations of seliciclib or CCT068127 for 4, 12, and 24 h and the effect on cell cycle distribution and DNA synthesis was assessed by incorporation of PI measured by flow cytometry (Fig. 3A). A modest reduction in the proportion of cells with 2n DNA content (~ 10%) and an increase in cells with 4n DNA content (~ 10%) are seen with both compounds (Fig. 3B). CCT068127 and seliciclib both blocked DNA synthesis, as determined by BrdU incorporation, although CCT068127 achieved this more rapidly than seliciclib and with 14‐fold greater potency (Fig. 3C). In both cases, the block in DNA synthesis is near complete at 24 h. Analysis of parallel treatments demonstrated a loss of RB phosphorylation as demonstrated by a mobility shift in the protein on SDS/PAGE (Fig. 3D).

### 3.5 Gene expression profiling of seliciclib analogs

**Figure 4:**
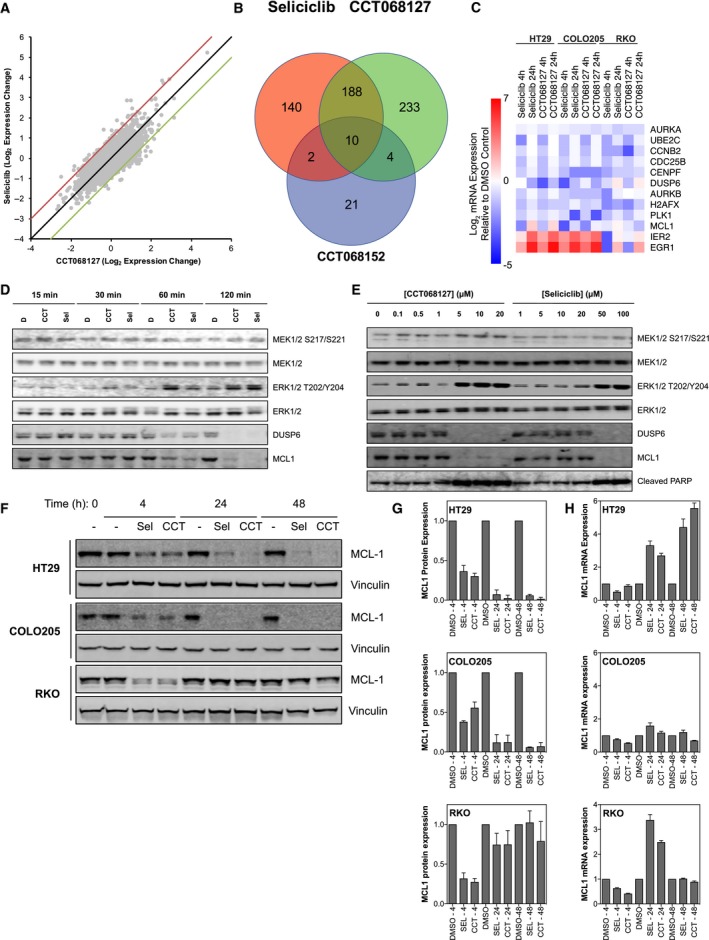
Gene expression analysis of colon cancer cells following treatment with CCT068127 and seliciclib. (A) HT29 cells were treated with equiactive concentrations (3xGI_50_, SRB assay) of seliciclib (45 μm) and CCT068127 (2.55 μm) or 45 μm CCT068152. mRNA expression profiles were generated using a custom microarray enriched for 5808 cDNAs implicated in cancer. Genes that were differentially expressed between the CDK inhibitor‐ and the DMSO‐treated cells were determined using comparative marker selection (*t*‐test) and a  ≥  twofold change in expression as a filter. The mRNA expression changes for seliciclib and CCT068127 were plotted against each other. (B) Venn diagram was constructed to show the number of genes in common between the two CDK inhibitors, and the inactive analog CCT068152, which displayed either increased or decreased expression following treatment. (C) mRNA expression changes following treatment of HT29, COLO205, or RKO human colon cancer cells with 3xGI_50_ concentrations of CCT068127 or seliciclib for 4 and 24 h as determined by RT‐qPCR. (D) HT29 cells were treated with 5xGI_50_ concentrations of CCT068127 (4.25 μm) or seliciclib (75 μm) for 15, 30, 60, or 120 minutes and cell lysates analyzed for the indicated proteins by western blotting. (E) HT29 cells were treated with a range of concentrations of CCT068127 or seliciclib for 24 h. Cells were lysed and extracts were analyzed by western blotting for the proteins listed. (F) HT29, COLO205, and RKO cells were treated with either DMSO or 3xGI_50_ concentrations of CCT068127 or seliciclib for 4, 24, and 48 h and protein lysates analyzed by western blotting for the indicated proteins. Data are representative of three independent repeats. (G) The expression of MCL1 in (F) was quantified and normalized to vinculin expression. Data are the mean of three independent repeats ± SE. (H) Cells were treated as in (F) and the expression of *MCL1* mRNA was quantified by qRT‐PCR, normalized to the housekeeping gene *GUSB*. The mean ± SE of triplicate measurements is presented.

In order to explore the potential similarities between seliciclib and CCT068127 in an unbiased manner, gene expression profiling was performed on HT29 human colon cancer cells treated with the respective 3xGI_50_ concentrations of the inhibitors for 24 h. As a control, cells were also treated with an inactive analog of seliciclib, CCT068152, and mRNA expression was measured. This compound displays no inhibitory activity against purified CDKs, has a GI_50_ > 50 μm on HT29 cells, and does not inhibit RB phosphorylation (Fig. S5). Comparison with this compound would potentially highlight any off‐target effects of the amino‐purine chemical backbone of the series. CCT068152 was used at an equimolar concentration when compared with seliciclib. Gene expression was determined using custom expression arrays (Whittaker *et al*., 2007). A table showing the transcriptional changes induced by each compound is presented in Table S4. When the gene expression changes induced by seliciclib and CCT068127 compound are plotted against each other, they show good concordance (Fig. 4A).

We found that seliciclib and CCT068127 share a core set of 198 genes (of a total of 577 affected) that are either increased or decreased in expression following treatment (Fig. 4B). This is consistent with the view that CCT068127 retains some of the same characteristics as seliciclib, although other effects may contribute. Of these shared 198 genes, the expression of only 10 is also altered by the inactive analog CCT068152, suggesting that these are likely off‐target effects, whereas the others are on‐target. In fact, we found that the inactive analog changes the expression of only 37 genes, markedly fewer than either seliciclib or CCT068127. For further analysis, genes that were altered in expression by CCT068152 were excluded.

The mRNA expression changes induced by treatment of HT29 cells with either seliciclib or CCT068127 were used to define a signature for each compound by identifying genes altered in expression by ≥ twofold following treatment. We then analyzed these signatures for similarity to known drug‐induced gene expression changes using the Connectivity Map (Lamb *et al*., 2006). Strikingly, signatures for both compounds show a high level of correlation with those of other small‐molecule CDK inhibitors, namely 0175029‐0000 (Khan *et al*., 2012), alsterpaullone (Schultz *et al*., 1999), and GW8510 (Bramson *et al*., 2001), as well as HDAC inhibitors vorinostat/SAHA (Richon *et al*., 1998), trichostatin A (Yoshida *et al*., 1990), valproic acid (Gottlicher *et al*., 2001) (Tables S5 and S6).

We also utilized the STRING database (Franceschini *et al*., 2013) to look for functional associations between genes with altered expression in response to CDK inhibition (Fig. S6). A cluster of genes involved in G_2_/M cell cycle progression is immediately evident, including decreased expression of several mitosis‐related genes, including *PLK1*,* CCNB2*,* AURKA*,* AURKB, CDC25B, UBE2C,* and *CENPF* (Fig. S6). These changes could bring about a G_2_/M‐phase arrest, which would be consistent with the 4n DNA population reflecting an increased G_2_/M phase and the reduced transcriptional output induced by the CDK inhibitors as we have previously characterized for seliciclib (Whittaker *et al*., 2007). We confirmed these gene expression changes by RT‐qPCR (Fig. 4C). Alternatively, this could also reflect a population of G_1_‐phase cells with 4n DNA content, which may arise through mitotic slippage, due to premature loss of CDK1/cyclin B activity (Rieder and Maiato, 2004).

As part of the overall core transcriptional profile determined by microarray (see above), we identified transcription factors downstream of the MAPK pathway whose expression is increased in response to the CDK inhibitors; these changes were also confirmed by RT‐qPCR (Fig. 4C). For example, the expression of the transcription factor‐encoding genes *EGR1* (coding for early growth response protein 1) and *IER2* (encoding immediate early response 2) was induced (Boros *et al*., 2009; Hodge *et al*., 1998). In contrast, the expression of *DUSP6—*encoding dual‐specificity phosphatase 6 that acts as a negative regulator of ERK2 by decreasing phosphorylation (Muda *et al*., 1996)—is reduced. Consistent with this, we have previously observed phosphorylation/activation of extracellular signal‐regulated kinases (ERKs) in response to seliciclib in human colon cancer cell lines (Whittaker *et al*., 2004). Our observation here of increased expression of transcripts regulated by the MAPK pathway was of particular interest as this suggested that activation of the pathway has functional consequences. We confirmed that treatment of HT29 cells with CCT068127 or seliciclib for 15, 30, 60, and 120 minutes results in rapid phosphorylation of ERK1/2 and that this is seen in the absence of any dramatic changes in the phosphorylation state of the upstream kinases MEK1 and MEK2 (Fig. 4D). Therefore, we determined the expression at the protein level of the ERK2‐phosphatase DUSP6, which as noted above shows reduced mRNA expression. We found that DUSP6 protein levels reduce rapidly, within 1–2 h following treatment with either CCT068127 or seliciclib, commensurate with increased ERK1/2 phosphorylation (Fig. 4D).

We also noted that CDK inhibitor treatment alters transcription of the antiapoptotic gene *MCL1,* which belongs to the BCL2 family. Although we observed an increase in *MCL1* mRNA at 24 h by microarray, RT‐qPCR analysis showed an initial decrease at 4 h, followed by a rebound to untreated levels or higher by 24 h (Fig. 4C). We found that MCL1 protein expression is decreased by both CCT068127 and seliciclib within 2 h of treatment (Fig. 4D) (Whittaker *et al*., 2007). Interestingly, unlike the mRNA, expression of MCL1 protein does not recover for 24 h with either seliciclib or CCT068127 (Fig. 4E). Sustained suppression of MCL1 protein expression by seliciclib and CCT068127 was confirmed in HT29 and COLO205 cells out to 48 h, whereas in RKO cells MCL1 protein was decreased at 4 h but recovered to near‐control levels at 24 and 48 h (Fig. 4F–H). qRT‐PCR of all of these cell lines treated as in Fig. 4F demonstrated that mRNA levels for MCL1 showed modest decreases at 4 h of treatment, followed by recovery to control levels or greater at 24 and 48 h. Notably, loss of MCL1 protein occurs at the same concentrations at which we also observe PARP cleavage—a marker of caspase activation and apoptosis (Fig. 4E). Hence, loss of MCL1 protein might present a deficit in the balance of antiapoptotic versus proapoptotic signals, resulting in the induction of apoptosis.

### 3.6 Combined inhibition of CDKs and the BCL2 family is synergistic

**Figure 5:**
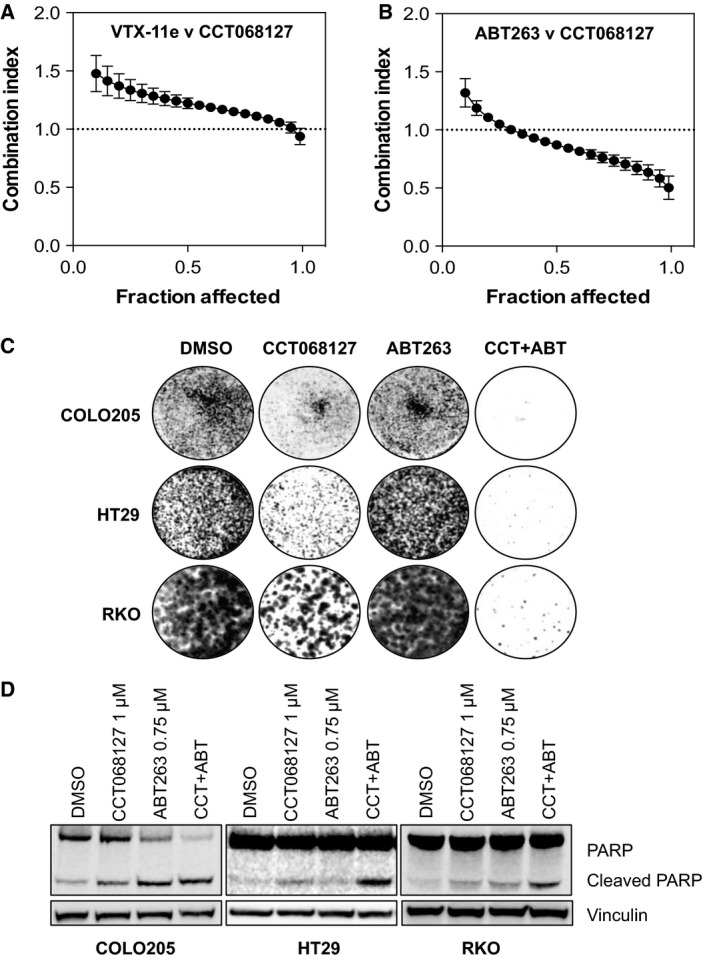
Targeting CDK2/9 and BCL2 family members synergistically inhibits proliferation and induces apoptosis. (A) HT29 human colon cancer cells were treated with increasing concentrations of CCT068127 in combination with a small‐molecule inhibitor of ERK, VTX‐11e. Cells were treated with both compounds in a 1.5‐fold dilution series starting at 5X the GI_50_ (4.25 μm CCT068127 and 243 nm VTX‐11e) in a 1 : 1 ratio. Cell proliferation/survival was assessed by SRB assay, and synergy was calculated using the combination index (CI) methodology (Chou and Talalay, 1984). Data from four independent repeats were analyzed by calcusyn software, and the mean CI ± SE is plotted relative to the fraction of cells affected by the treatment. Values < 1 are indicative of synergy. (B) HT29 cells were treated as in (A) except the ERK inhibitor was replaced by the BCL2 family inhibitor ABT263. Cells were treated with both compounds in a 1.5‐fold dilution series starting at 5X the GI_50_ (4.25 μm CCT068127 and 12.7 μm ABT263) in a 1 : 1 ratio. The mean combination index ± SE is plotted relative to the fraction of cells affected by the treatment. Values < 1 are indicative of synergy. (C) HT29, COLO205, and RKO human colon cancer cells were treated with 1 μm CCT068127 or 750 nm ABT263 alone, or in combination for 5 days after which the medium was replaced and cells were cultured in medium alone for a further 7 days. Cells were then fixed and stained with crystal violet. Data shown are representative of three independent experiments. (D) HT29, COLO205, and RKO cells were treated with 1 μm CCT068127 in the presence and absence of 750 nm ABT263 for 48 h. Cell lysates were analyzed by western blotting for the indicated proteins.

Our observation that CCT068127 treatment gives rise to enhanced MAPK signaling prompted us to hypothesize that activation of the MAPK pathway might provide a survival signal to the cells. We therefore treated HT29 human colon cancer cells with a selective inhibitor of ERK known as VTX‐11e (Aronov *et al*., 2009) in combination with CCT068127; we then determined the effect on cell proliferation and survival after 4 days and calculated synergy using a well‐established method (Chou and Talalay, 1984). Under these conditions, the ERK inhibitor plus CCT068127 gave combination index (CI) values of >1 and was classified as antagonistic (Fig. 5A).

As we also observed a loss of the antiapoptotic MCL1 protein in response to CDK inhibitor treatment, we posited that this depletion of MCL1 might create a dependency upon other antiapoptotic proteins, such as additional members of the BCL2 family, and that a BCL2 family inhibitor might be synergistic with CCT068127. Hence, we investigated the response of HT29 cells to a 96‐h exposure to the combination of CCT068127 and ABT263, an inhibitor of BCL2 family proteins (Tse *et al*., 2008). Excitingly, we observed with a CI value ~0.5 or lower, indicating a synergistic antiproliferative effect with the combination of these two agents (Fig. 5B). We confirmed that the combination of these two agents results in an increased antiproliferative/cytotoxic effect as measured by colony formation assay in HT29, COLO205, and RKO cells (Fig. 5C). Colorectal cancer cells were exposed to 1 μm CCT068127, 750 nm ABT263, or a combination of both drugs for a period of 5 days. The drugs were then washed out and the cells grown in medium only for a further 7 days. We observed that the combination of CCT068127 and ABT263 results in a significantly greater suppression of measurable cell colony formation than when used as single agents (Fig. S7). Furthermore, exposure of HT29, COLO205, and RKO human colon cancer cells to both compounds in combination for 48 h results in a greater degree of PARP cleavage, indicative of apoptotic cell death (Fig. 5D).

## 4 Discussion

We set out to discover a CDK2/9 inhibitor with greater potency and metabolic stability compared with the parent compound seliciclib (Wilson *et al*., 2011). CCT068127 exhibits 15‐fold greater potency against CDK1/cyclin B, 14‐fold greater potency against CDK5/p35, 22‐fold greater potency against CDK2/cyclin E, and 11‐fold greater potency against CDK9/cyclin T. In contrast, potency toward CDK4 and CDK6 increased only by 6‐ to 8‐fold and that for CDK7/cyclin H potency remained the same; therefore, selectivity for CDK2 and 9 was increased over CDKs 4, 6, and 7. Despite increased potency against CDK1, a cell‐based assay for PP1 phosphorylation at T320 demonstrated that CDK1 was not inhibited at concentrations of CCT068127 (or seliciclib) where cellular effects on proliferation and signaling occurred. The basis for greater potency against CDK2 was suggested by the crystal structure of CCT068127 bound to CDK2, whereby the pendant C2 side chain hydroxyl moiety forms an additional hydrogen bond with Asp145 of the DFG motif. Improved enzyme potency translated into greater antiproliferative activity against human cancer cell lines with an average GI_50_ 24‐fold lower than that observed with seliciclib. Importantly, there was a clear correlation between the suppression of key pharmacodynamic markers for CDK2 and CDK9 (phosphorylation of RB and RNA polymerase II, respectively) and the inhibition of cell proliferation. Notably, RNA polymerase II was inhibited more rapidly and near completely compared to RB by both seliciclib and CCT068127, suggesting that inhibition of CDKs targeting RNA polymerase II may be a primary driver of the cellular response to these agents. This is consistent with a recent study in which seliciclib was shown to bind to CDK7, 12, 9, and 2 in an affinity pull‐down assay (Delehouze *et al*., 2014). The consequence of CDK9 inhibition appears to be a gradual reduction in the expression of cyclin D1, cyclin T1, and CDK9 itself presumably via inhibition of transcriptional elongation by RNA polymerase II. Thus, these agents may primarily target CDKs that regulate transcription but, through cyclin or CDK depletion, result in inhibition of additional cell cycle CDKs as well. While *in vitro* biochemical data suggest that CCT068127 showed greatest potency for CDK2, CDK5, and CDK9, our cellular studies suggest that CDK9 may be a key target of this compound, supported by the key observations of the greater magnitude of inhibition of RNA polymerase II phosphorylation over RB, that the catalytic subunit of CDK5 was not detectable in the HT29 and RKO cell lines, and that siRNA‐mediated reduction in CDK5 expression did not have marked effects on HT29 cell proliferation. However, we cannot rule out the possibility that inhibition of these, and potentially other CDKs, may contribute to the mechanism of action of these compounds or additional effects *in vivo*.

In terms of cell cycle effects, CCT068127 caused the same arrest of cell cycle progression as seen with seliciclib, with a reduction in cells with 2n DNA content and an increase in cells with 4n DNA content, but was able to achieve this at a concentration 15‐fold lower than that required for seliciclib. Overall, CCT068127 retained the same cellular effects as the developmental clinical drug seliciclib but with significantly improved potency and, as we have reported previously, has improved metabolic stability and displays greater *in vivo* efficacy in a tumor xenograft model (Wilson *et al*., 2011).

We further investigated the mechanism of action of CCT068127 by comparing the transcriptional signature induced by the compound in the HT29 human colon cancer cell line to the expression signatures available in the Connectivity Map database. The signatures most highly correlated with that of CCT068127 are those for other CDK inhibitors and, in addition, HDAC inhibitors. The close correlation between these two compound classes may stem from their ability to broadly modulate transcription and that functional RB recruits histone deacetylase to suppress E2F‐regulated transcription (Brehm *et al*., 1998), which is enhanced by CDK inhibition. More global effects on transcription are also likely to be a result of decreased RNA polymerase II activity, through inhibition of CDKs including CDK9.

It is interesting to note that treatment with CCT068127 induces rapid activation of ERK1/2 and we were keen to determine how this was mediated and whether there was an associated dependency upon MAPK signaling for survival of the cells. As the phosphorylation state and expression of MEK1/2 appeared to be unaltered by CCT068127 treatment, our results suggested that upstream activation of the MAPK pathway was likely not mediating this effect. In fact, decreased protein expression of the ERK phosphatase DUSP6 occurs at the same time and concentrations of CCT068127 as the observed induction of ERK1/2 phosphorylation. It therefore seems likely that loss of DUSP6 expression permits increased ERK1/2 phosphorylation and activation of the pathway (Muda *et al*., 1996). Consistent with this, the mRNAs for EGR1 and IER2 were induced by both inhibitors, suggesting some capacity of ERK to positively modulate transcription. Given the positive regulation of cell proliferation and survival mediated by the MAPK pathway, we hypothesized that addition of an ERK inhibitor would block any proliferative or survival signaling through MAPK. Surprisingly, this was not the case and rather than showing synergy with CCT068127, the ERK inhibitor was antagonistic. ERK inhibition reportedly induces a G_1_ arrest (Morris *et al*., 2013) and therefore could potentially protect cells from CDKi‐mediated apoptosis, particularly if driven by deregulation of E2F during S phase (Qin *et al*., 1994; Wu and Levine, 1994).

The mRNA expression profiling data showed an increase in the transcript levels of the antiapoptotic gene *MCL1* in the HT29 cell line. Previously, we had seen this with seliciclib treatment but the expression of MCL1 protein was rapidly decreased in response to CDK inhibition (Whittaker *et al*., 2007) as has been described for several other CDK9 inhibitors (Gregory *et al*., 2015; Lemke *et al*., 2014; Ma *et al*., 2003). Further RT‐qPCR analysis showed that *MCL1* mRNA was initially inhibited in all three cancer cell lines at 4 h but was substantially increased in HT29 and RKO cells by both seliciclib and CCT068127 at 24–48 h. However, MCL1 protein levels were decreased by both compounds at 4–48 h in HT29 and COLO205 cells, whereas RKO cells showed a decrease at 4 h and recovery at 24–48 h. Given the effect of CDK2 and CDK9 inhibition on the cell cycle and global transcriptional regulation, presumably sustained MCL1 protein suppression at 24–48 h is due to either inhibition of translation of the mRNA or accelerated protein degradation (Choudhary *et al*., 2015). Notably, the RKO cells recover expression of MCL1 protein at 24–48 h so may regulate MCL1 protein expression differently to the HT29 and COLO205 cells. Irrespective of the precise mechanism, the loss of MCL1 protein presented a possible therapeutic opportunity to further target the antiapoptotic BCL2 family with a small‐molecule inhibitor and enhance the proapoptotic activity of CCT068127. Combined use of CCT068127 and ABT263, a BCL2 family inhibitor, led to synergistic antiproliferative activity and consistent with this, we saw elevated caspase activation indicative of apoptosis and reduced cell survival in colony formation assays. Excitingly, this presents a highly rational and mechanistically defined combinatorial approach to target cancer cell survival. Given that *MCL1* is amplified in approximately 11% of cancers and overexpression of the protein is associated with chemoresistance (Beroukhim *et al*., 2010), the combination of a CDK2/9 inhibitor with a BCL2 family inhibitor presents an attractive therapeutic strategy for those cancers that may have an increased dependency on MCL1. To our knowledge, this is the first demonstration that combination of a CDK2/9 inhibitor and a BCL2 family inhibitor is beneficial in human colorectal cancer cell lines and may support widening the scope of indications in which these agents are tested. Overall, our data support the continued clinical development of this series of CDK2/9 inhibitors, from which CYC065 has now entered first‐in‐human studies (Cyclacel Ltd. press release October 22, 2015).

## Conflict of interests

The Institute of Cancer Research has a commercial interest in the development of CDK inhibitors and operates a reward for discoverers scheme. PW has been or is a consultant to Novartis, Astex Pharmaceuticals, Chroma Therapeutics, Piramed Pharma, Nuevolution, and NexTech Ventures.

## Author contributions

SRW, CB, CM, SWa, AS, EB, SS, RTP, and PAC carried out cell‐based studies including cell proliferation assays, RT‐qPCR, western blotting, microarray analysis, and colony formation assays. NB and JB conducted and interpreted *in silico* docking studies of CCT068127 and seliciclib. MPM and MN generated and analyzed the cocrystal structure of CCT068127 and CDK2/cyclin A. SWi and EMcD designed and synthesized CCT068127. WJ and PMF contributed to the biochemical characterization and development of CCT068127. SRW, PAC, MIW, JB, MDG, and PW were responsible for study conception, design, and data interpretation. SRW, MPM, MN, JB, and PW wrote the manuscript.

## Supporting information


**Fig. S1.** Inhibition of CDK1 activity in HT29 colon cancer cells treated with CDK inhibitors.

**Fig. S2**. CCT068127 is a potent inhibitor of RNA polymerase II phosphorylation in COLO205 cells.

**Fig. S3**. CCT068127 treatment decreases the expression of cyclin T1, CDK9 and cyclin D1.

**Fig. S4**. CDK5 is not required for cell proliferation.

**Fig. S5.** CCT068152 is an inactive analogue of CCT068127.

**Fig. S6.** Gene expression profiling of seliciclib and CCT068127 identifies a cluster of genes involved in G_2_/M cell cycle control.

**Fig. S7.** HT29, COLO205 and RKO human colon cancer cells were treated with 1 μm CCT068127 or 750 nm ABT263 alone, or in combination for 5 days after which the medium was replaced and cells were cultured in medium alone for a further 7 days.

**Table S1.** Primary antibodies used, supplier, catalogue number, species and protein molecular weight.
**Table S2.** Primer sequences used for qRT‐PCR assays.
**Table S3.** Summary of Crystallographic Data Collection and Structure Refinement.
**Table S4.** mRNA expression data for HT29 cells treated with 3xGI50 concentrations of seliciclib, CCT068127 or CCT068152 for 24 h. Probe intensities were log2 normalised.
**Table S5.** CMAP correlations of gene expression changes induced by CCT068127.
**Table S6.** CMAP correlations of gene expression changes induced by seliciclib.
