# Supplementary figures and images for: Molecular profiling and combinatorial activity of CCT068127: a potent CDK2 and CDK9 inhibitor
Source: Mol Oncol. 2018 Jan 28;12(3):287–304. doi: 10.1002/1878-0261.12148 (PMC5830651; doi:10.1002/1878-0261.12148)

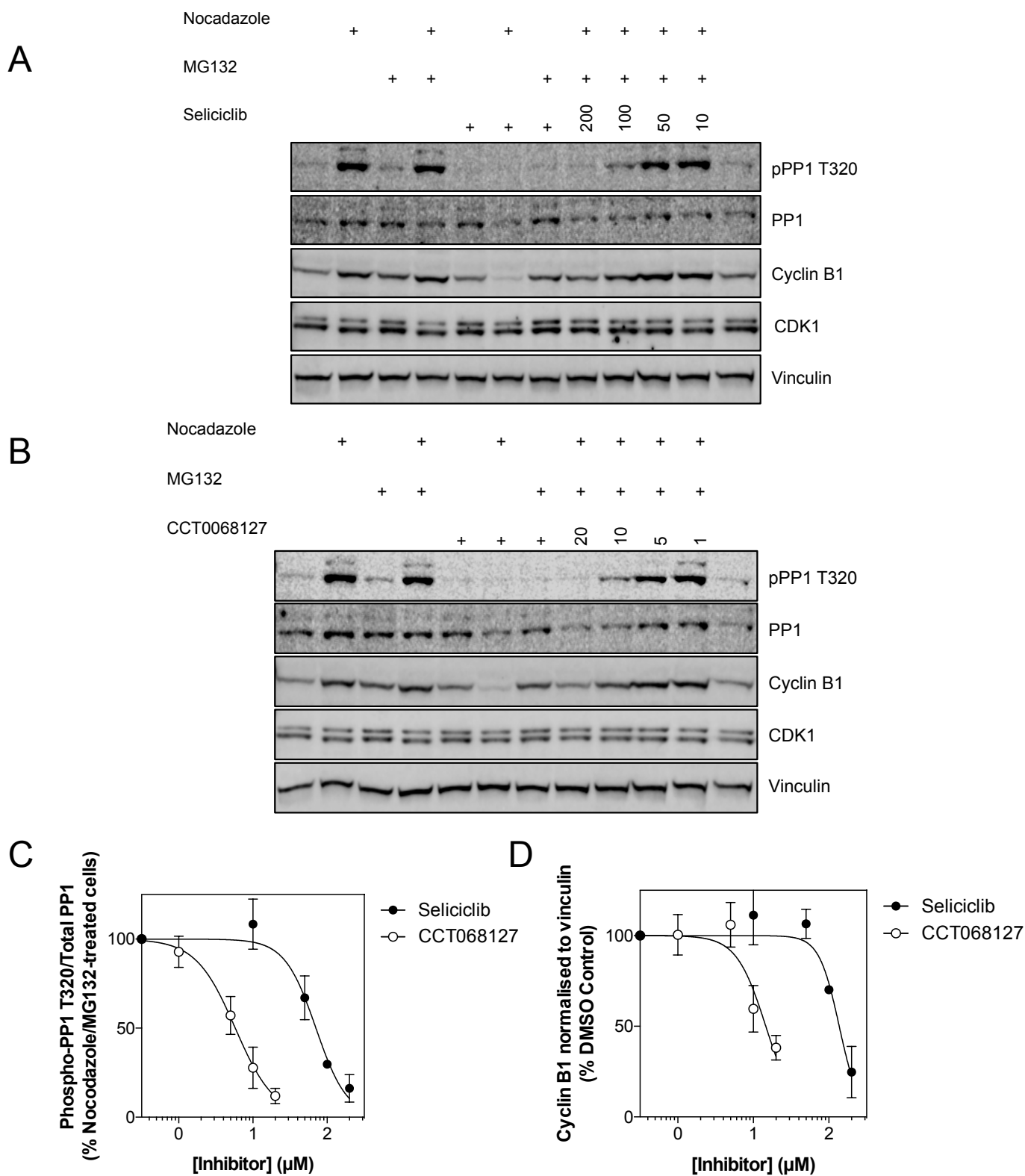

Supplementary Figure 1. Whittaker et al.

Supplement: Supplementary file 1 — Fig. S1. Inhibition of CDK1 activity in HT29 colon cancer cells treated with CDK inhibitors. [file MOL2-12-287-s001.pdf]

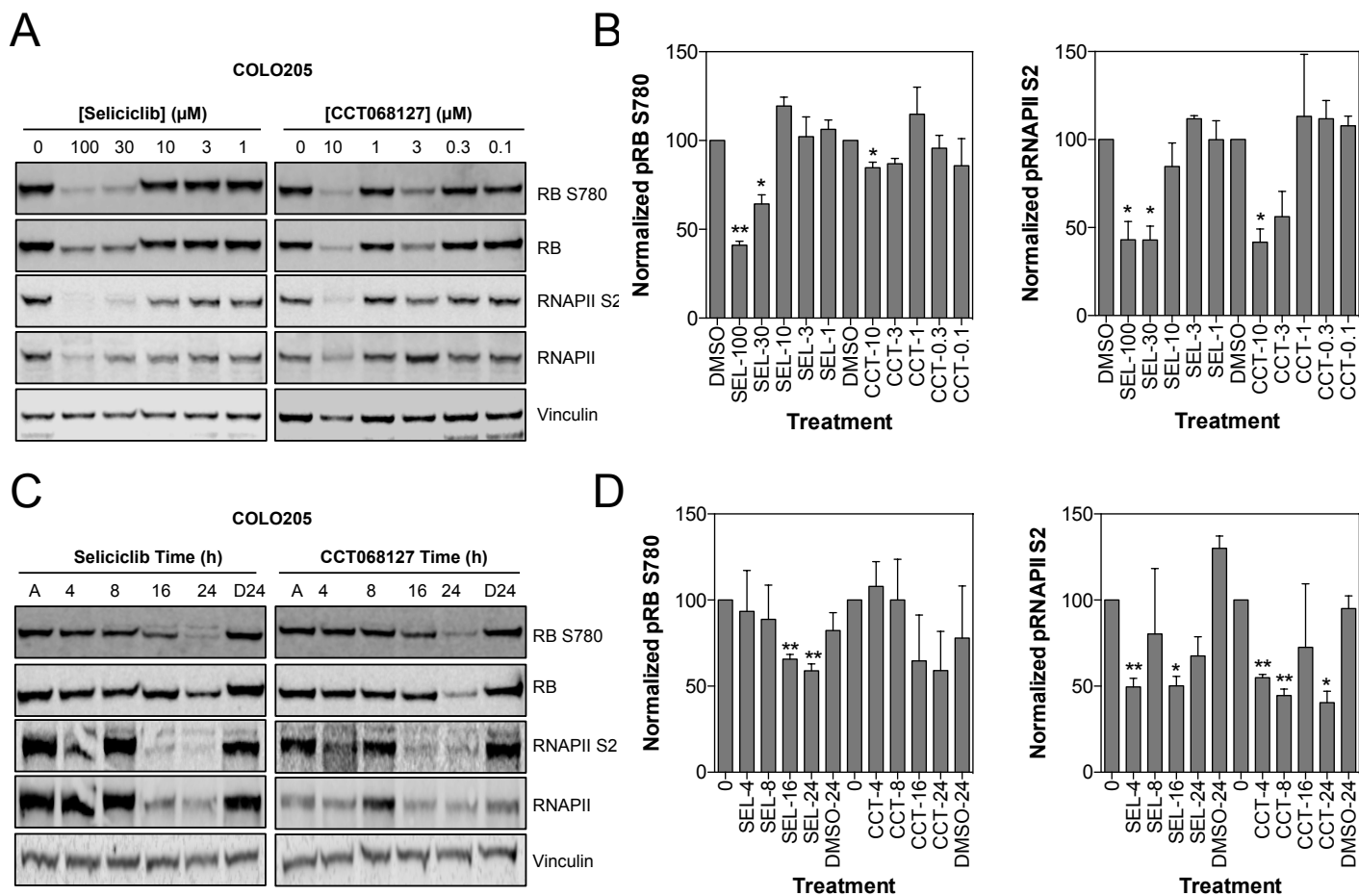

Supplementary Figure 2. Whittaker et al.

Supplement: Supplementary file 2 — Fig. S2. CCT068127 is a potent inhibitor of RNA polymerase II phosphorylation in COLO205 cells. [file MOL2-12-287-s002.pdf]

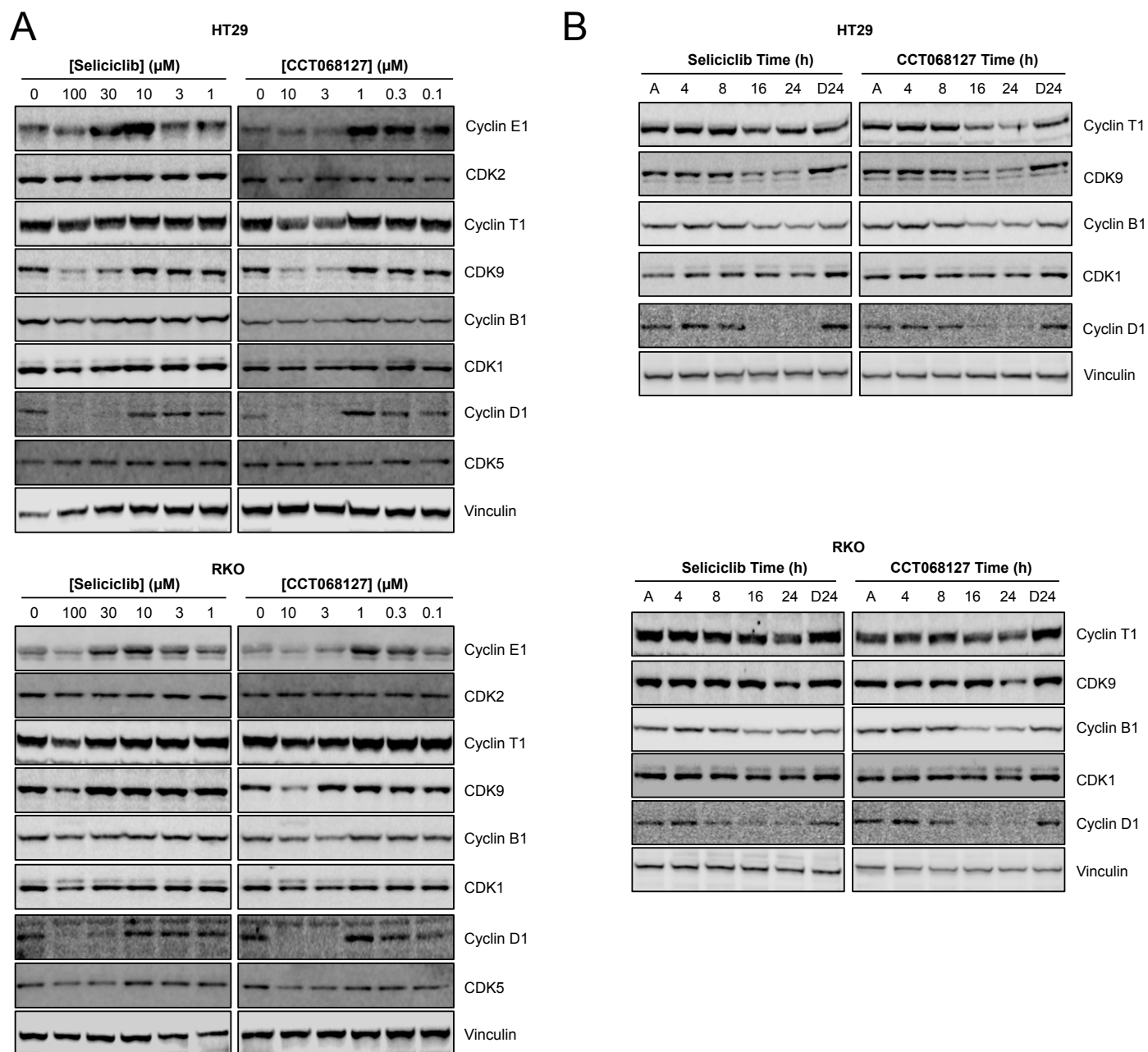

Supplementary Figure 3. Whittaker et al.

Supplement: Supplementary file 3 — Fig. S3. CCT068127 treatment decreases the expression of cyclin T1, CDK9 and cyclin D1. [file MOL2-12-287-s003.pdf]

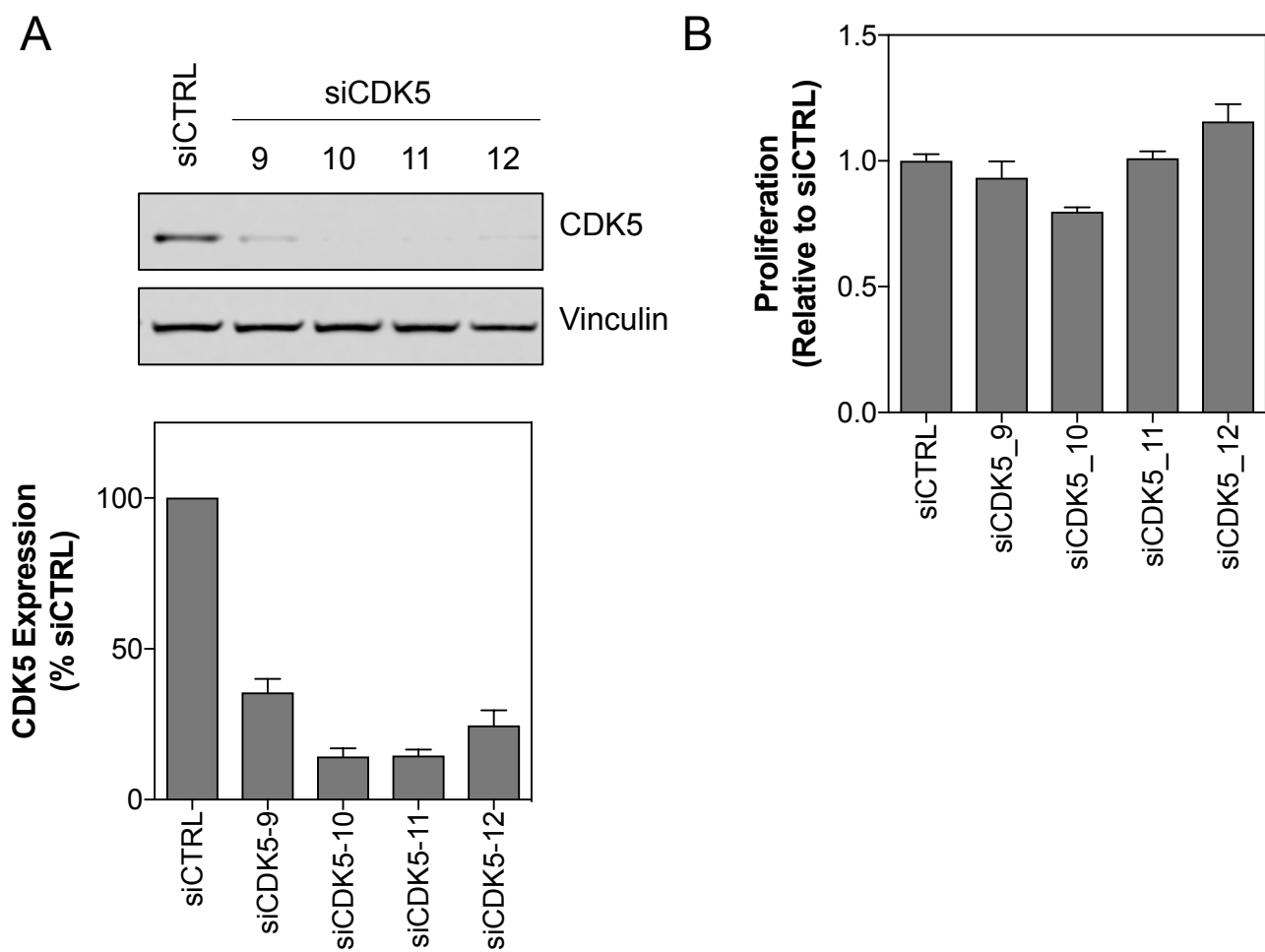

Supplementary Figure 4. Whittaker et al.

Supplement: Supplementary file 4 — Fig. S4. CDK5 is not required for cell proliferation. [file MOL2-12-287-s004.pdf]

A

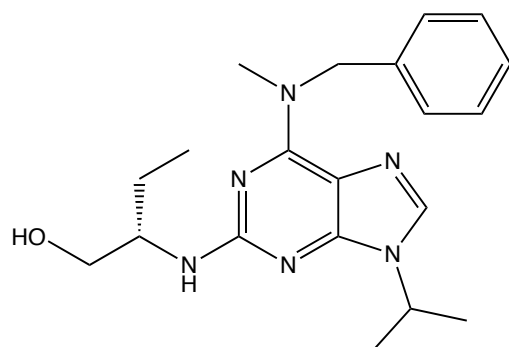**CCT068152**

B

| Kinase  | IC <sub>50</sub> (μmol/L) |
|---------|---------------------------|
| CDK1/B1 | >200                      |
| CDK2/E  | >50                       |
| CDK4/D1 | >200                      |
| CDK7/H  | >200                      |
| ERK2    | >200                      |

C

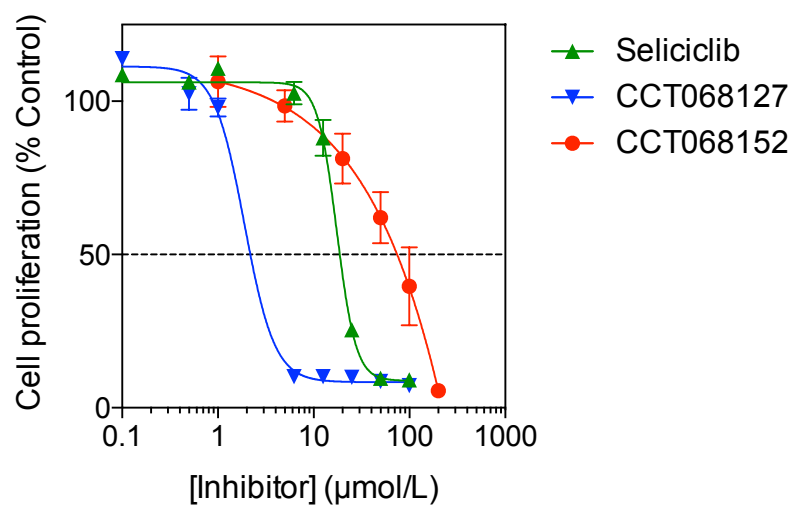

D

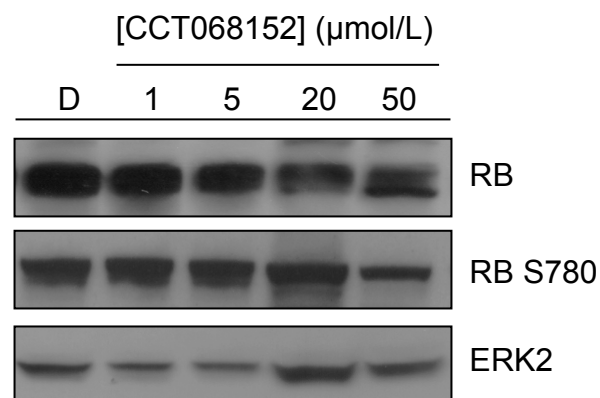

Supplement: Supplementary file 5 — Fig. S5. CCT068152 is an inactive analogue of CCT068127. [file MOL2-12-287-s005.pdf]

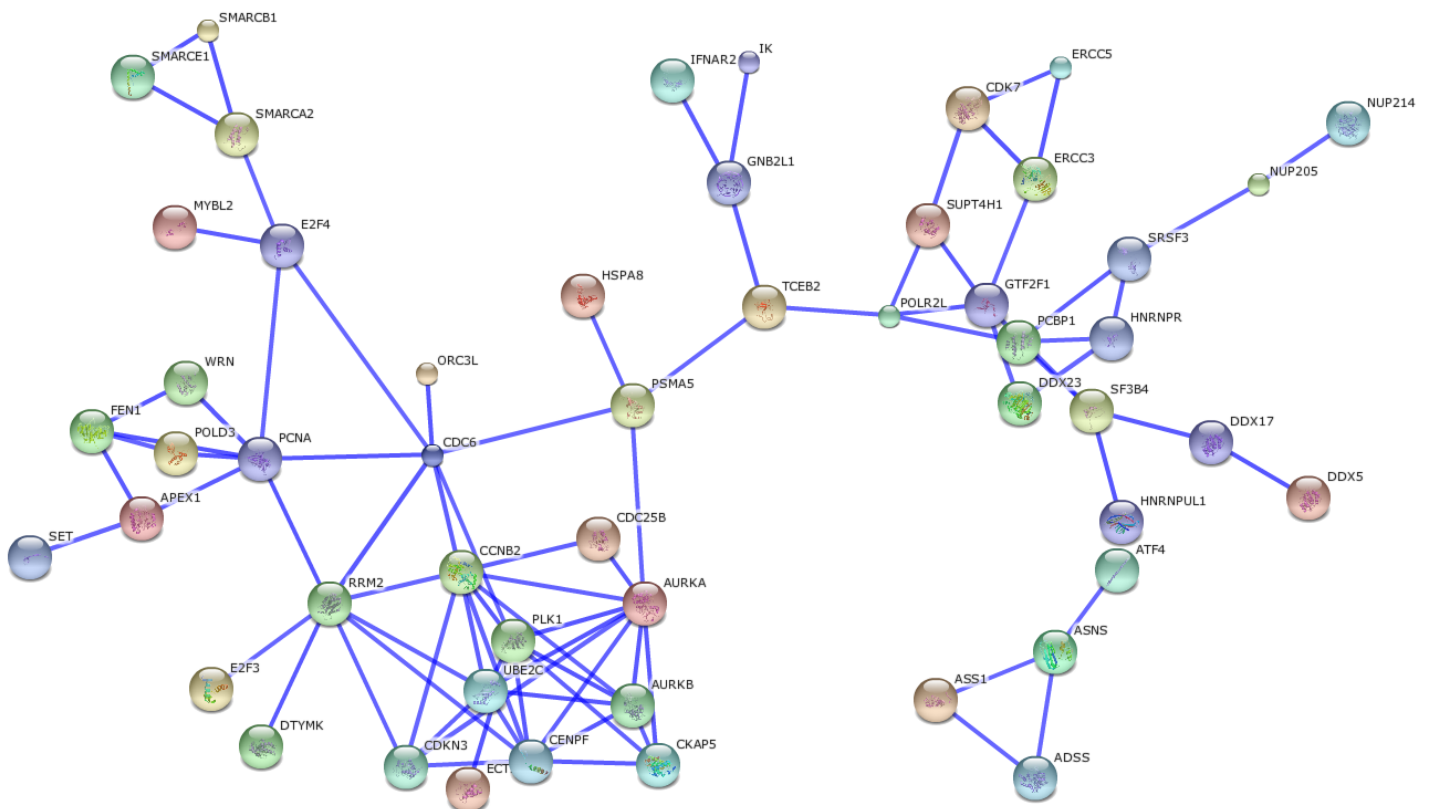

**Supplementary Figure 6. Whittaker et al.**

Supplement: Supplementary file 6 — Fig. S6. Gene expression profiling of seliciclib and CCT068127 identifies a cluster of genes involved in G2/M cell cycle control. [file MOL2-12-287-s006.pdf]

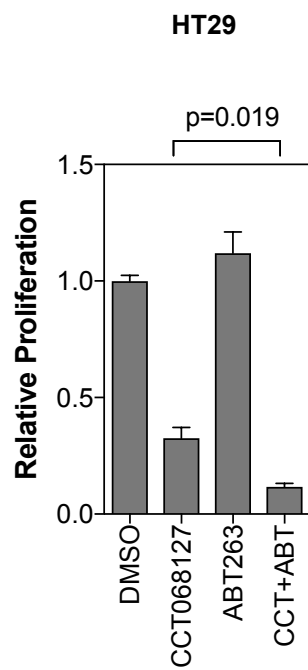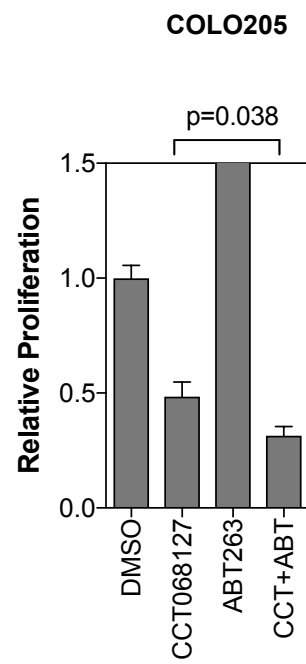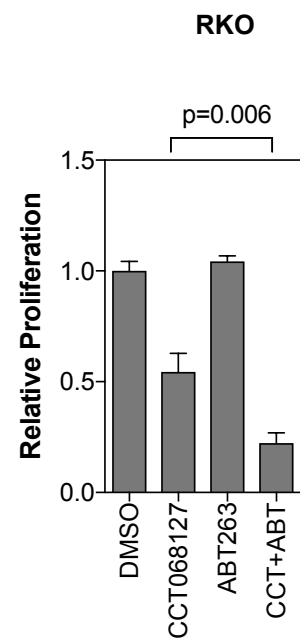

Supplementary Figure 7, Whittaker et al.

Supplement: Supplementary file 7 — Fig. S7. HT29, COLO205 and RKO human colon cancer cells were treated with 1 μm CCT068127 or 750 nm ABT263 alone, or in combination for 5 days after which the medium was replaced and cells were cultured in medium alone for a further 7 days. [file MOL2-12-287-s007.pdf]
